# Exploitation of semantic methods to cluster pharmacovigilance terms

**DOI:** 10.1186/2041-1480-5-18

**Published:** 2014-04-16

**Authors:** Marie Dupuch, Laëtitia Dupuch, Thierry Hamon, Natalia Grabar

**Affiliations:** 1 CNRS UMR 8163 STL; Université Lille 1&3, F-59653 Villeneuve d’Ascq, France; 2 Centre de Recherche des Cordeliers, Université Pierre et Marie Curie - Paris6, UMR_S 872, Paris F-75006, France; 3 INSERM, U872, Paris F-75006 France; 4 Université Toulouse III Paul Sabatier, F-31062 Toulouse, France; 5 LIMSI-CNRS, BP133 Orsay, France; 6 Université Paris 13, Sorbonne Paris Cité, France

## Abstract

Pharmacovigilance is the activity related to the collection, analysis and prevention of adverse drug reactions (ADRs) induced by drugs. This activity is usually performed within dedicated databases (national, European, international...), in which the ADRs declared for patients are usually coded with a specific controlled terminology MedDRA (Medical Dictionary for Drug Regulatory Activities). Traditionally, the detection of adverse drug reactions is performed with data mining algorithms, while more recently the groupings of close ADR terms are also being exploited. The Standardized MedDRA Queries (SMQs) have become a standard in pharmacovigilance. They are created manually by international boards of experts with the objective to group together the MedDRA terms related to a given safety topic. Within the MedDRA version 13, 84 SMQs exist, although several important safety topics are not yet covered. The objective of our work is to propose an automatic method for assisting the creation of SMQs using the clustering of semantically close MedDRA terms. The experimented method relies on semantic approaches: semantic distance and similarity algorithms, terminology structuring methods and term clustering. The obtained results indicate that the proposed unsupervised methods appear to be complementary for this task, they can generate subsets of the existing SMQs and make this process systematic and less time consuming.

## Introduction

The development of new drugs has allowed the treatment of many diseases that were previously considered incurable and with potential fatal outcomes for patients. However, this major therapeutic advance is limited by the toxicity of some drugs that may also be dangerous for patients. To minimize the risks associated with drug use, it is necessary to detect as early as possible the adverse drug reactions (ADRs) that may have been unnoticed during clinical trials. This is the role of regulatory authorities and of pharmacovigilance units within pharmaceutical laboratories and hospitals. The main source of knowledge for pharmacovigilance is based on the reporting of the ADRs by health professionals and patients. These case reports are recorded in pharmacovigilance databases. To facilitate the analysis of those data, ADRs are coded using a controlled vocabulary, usually MedDRA (Medical Dictionary for Drug Regulatory Activities) [1]. The detection of new pharmacovigilance alerts, or signal detection, is typically based on a manual review of case reports by experts, and more recently in some countries by data mining techniques [2,3]. MedDRA is a fine-grained vocabulary with over 80,000 terms and it has been shown that the grouping of similar MedDRA terms (*i.e.*, *Hepatitis infectious*, *Hepatitis infectious mononucleosis*, *Hepatitis viral*) is often necessary in the process of the signal detection [4,5]. It may allow indeed to detect the toxicity of a drug more quickly.

The MedDRA terms are structured into five hierarchical levels (Table 1). From the highest to the lowest, these levels are: System organ class (SOC), High level group term (HLGT), High level term (HLT), Preferred term (PT), and Low level term (LLT). The hierarchical organization of the MedDRA terminology is clearly oriented on the division by organ system, *i.e.* among the SOCs we can find for instance *Musculoskeletal and connective tissue disorders*, *Hepatobiliary disorders*, *Psychiatric disorders* and *Cardiac disorders*. In Table 1, we indicate also examples of terms belonging to these five hierarchical levels. In the majority of cases, hierarchical levels have the subsumption is-a relations between them. For instance, in Table 1, the PT *Arthritis*is-a HLT *Arthropathies NEC*, while the HLT *Arthropathies NEC*is-a HLGT *Joint disorders*. The situation is different when we consider the relations between PTs and their LLTs [6]: these are no more subsumption relations but identical or subsumption relations instead, as the LLTs may be synonym or subordinate to their PTs. Thus, in Table 1, the LLT *Arthritis* is identical to its PT term *Arthritis*, although other LLTs such as *Arthritis aggravated, Atrophic arthritis, Joint inflammation, Finger arthritis, Knee arthritis* are subordinated to this PT.

**Table 1:** Structure of MedDRA: five hierarchical levels of MedDRA, number of terms per level and some examples of the terms

**Level**	**Expanded form**	**Nb terms**	**Examples**
SOC	System Organ Class	26	*Musculoskeletal and connective tissue disorders*
HLGT	High Level Group Terms	332	*Joint disorders*
HLT	High Level Terms	1,688	*Arthropathies NEC*
PT	Prefered Terms	18,209	*Arthritis*
LLT	Lowest Level Terms	66,587	*Arthritis, Arthritis aggravated, Atrophic arthritis*
			*Joint inflammation, Finger arthritis*
Total		86,842	

A first method to group the MedDRA terms is based on the hierarchical levels in MedDRA: HLT (High Level Terms), HLGT (High Level Group Terms) or SOC (System Organ Class) [7,8]. However, it was observed that some safety topics are orthogonal to these hierarchical levels (their terms may belong to different SOCs), which led to the development of the Standardized MedDRA Queries (SMQs) containing the MedDRA terms in connection with a safety topic [9] and independently from their SOCs. For example, the *Haemorrhage SMQ* contains MedDRA terms related to bleeding in all parts of the body: it groups terms from a large set of SOCs. The SMQs are developed internationally by experts looking manually in all the MedDRA terms relevant to each SMQ. There are currently 84 SMQs that do not cover the entire drug-induced set of safety topics (*Haemorrhage, Hepatic disorders, Systemic lupus erythematosus, Convulsions...*). This situation leads us to propose methods for automating the clustering of the terms when MedDRA provides no grouping category appropriate for a given safety topic. The lists of MedDRA terms may then be presented for the selection to the experts.

Other work on the automatic clustering of pharmacovigilance terms relies on a specific resource ontoEIM. ontoEIM stands for *ontology* and *ÉvénementsIatrogènesMédicamenteux* (Adverse Drug Effects in French) [10]. This resource is created through the projection of MedDRA on the terminology SNOMED CT (Systematized Nomenclature of Medicine - Clinical Terms) [11]. The projection is performed on the basis of the UMLS (Unified Medical Language System) [12], in which several terminologies are already merged and aligned, including MedDRA and SNOMED CT. The ontoEIM resource has been exploited to build groupings through the hierarchical subsumption [10,13]. Precision observed is high while the recall is extremely low, which is due to the fact that the SMQs contain terms from different SOCs. In other experiments, the ontoEIM resource has been used in combination with the semantic distance algorithms and applied to a subset of the MedDRA terms [14]. The same approach has been applied to a subset of WHO-ART (WHO Adverse Reaction Terminology) terms [15]. In the WHO-ART related experiment, the obtained groupings demonstrated interesting results because several types of semantic relations were detected between the terms (synonyms, antonyms, physiological functions or abnormalities, associated symptoms, abnormal laboratory tests, pathologies and their causes, close anatomical localization, degrees of severity, and heterogeneous groupings), although these groupings were not compared with the SMQs.

## Objectives

We address the problem of grouping the MedDRA pharmacovigilance terms in a way that reflects coherent and medically sound safety topics. Although the MedDRA vocabulary is structured according to specific organ-based semantic characteristics of the terms, this organization does not fully capture important semantic relationships among terms. We aim to explore how to group these terms in a way that directly reflects the intuitions captured in manually created SMQs. More precisely, our objective is to work on semantic methods for the automatic creation of groupings of the MedDRA terms. We propose to adapt and to combine two strategies: semantic distance and similarity algorithms, and terminology structuring methods. Special attention is paid to the merging and comparison between these two methods and evaluation of the generated term clusters. In order to measure the ability of our methods to produce clusters similar to the existing SMQs, we evaluate the generated clusters by taking these existing SMQs as a gold standard. Our method relies on two main assumptions: (1) the MedDRA terms can be used for the automatic creation of groupings of terms; (2) the combination of the semantic methods provide complementary results.

## Methods

**Figure 1:**
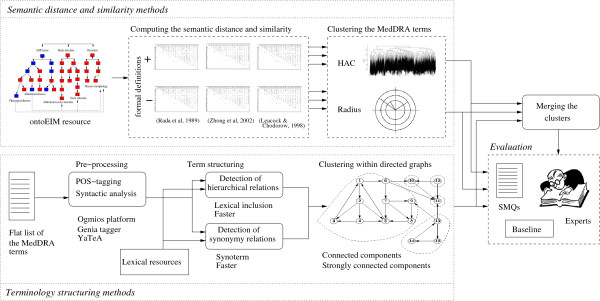
**General schema of the method.** Figure 1 presents the general schema of the proposed methods. The methods consist into four main steps: application of the semantic similarity and distance methods, application of the terminology structuring methods, clustering of the semantically similar terms and evaluation of the obtained results.

The method is organized in four main steps (Figure 1): (1) computing the semantic distance and similarity between the MedDRA terms using ontoEIM, (2) computing the semantic relations from a flat list of the MedDRA terms with the terminology structuring methods, (3) clustering the MedDRA terms, (4) and evaluating the obtained clusters against the SMQs. For the implementation, we exploit Perl and *R* (http://www.r-project.org) languages, and several Natural Language Processing (NLP) tools.

### Data

We make use of several types of material.

#### MedDRA terms

**Figure 2:**
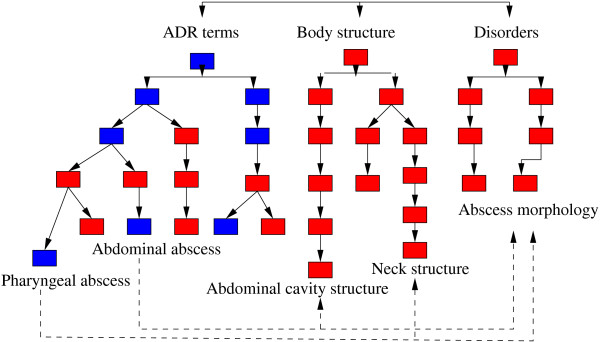
**Computing the shortest paths between two terms.** Figure 2 presents the principle for the computing of the shortest paths *sp* between two MedDRA terms (*Abdominal abscess* and *Pharyngeal abscess*) and between the elements of their formal definitions (axis Disorders and Body structure). Blue nodes are inherited from MedDRA, red nodes from SNOMED CT.

The MedDRA PT terms (n = 18,209) are exploited either as a flat list of terms, in which case the semantic relations between them are computed with terminology structuring methods, or through the ontoEIM resource [10], in which case the semantic relatedness between them is computed with semantic distance and similarity algorithms. We work with the *PT* terms because they are used for building the SMQs and for coding the pharmacovigilance case reports. The ontoEIM resource attempts to improve the MedDRA structuring in two ways: the structure of MedDRA terms becomes similar to the structure in SNOMED CT which makes it more fine-grained (the hierarchy is modified and enriched, and contains up to 14 hierarchical levels); and the MedDRA terms receive formal definitions (decomposition into their semantic primitives). Thus, in Table 2, the MedDRA ADR terms *Abdominal abscess* and *Pharyngeal abscess* are defined on two axes (Disorders and Body structure). For instance, *Pharyngeal abscess* is semantically decomposed into the Disorder element *Abscess morphology* and Body structure element *Neck structure*. The names of the formal definition elements correspond to the names of the hierarchies of the SNOMED CT. Within the ontoEIM, we have three hierarchical trees (Figure 2): one for the MedDRA terms and one for each axis of the formal definitions. The ontoEIM resource is used with the semantic similarity and distance algorithms.

**Table 2:** **Example of a formal definition for the MedDRA terms** ***Abdominal abscess* and** ***Pharyngeal abscess***

***MedDRA terms***	***Disorders***	***Body structure***
	**Type of abnormality**	**Anatomical localization**
*Abdominal abscess*	Abscess morphology	Abdominal cavity structure
*Pharyngeal abscess*	Abscess morphology	Neck structure

The terms are semantically decomposed into their elements from Disorders and Body structure axes.

#### Lexical resources

Three kinds of lexical resources are involved in the methods: (1) synonyms extracted from the UMLS (n = 228,542); (2) synonyms acquired from three biomedical terminologies thanks to their compositionality [16] (n = 28,691); (3) synonyms from WordNet [17] (n = 45,782). Lexical resources provide pairs of synonyms such as {*accord, concordance*}, {*pain, ache*}, {*aceperone, acetabutone*}, {*adenazole tocladesine*} or {*bleeding, haemorrhage*}. These resources are used with the terminology structuring methods.

#### Standardized MedDRA Queries (SMQs)

We use the 84 SMQs (2010 version) as the gold standard for the evaluation of the generated clusters of terms. The SMQs contain MedDRA terms relevant to a given safety topic. These terms usually belong to different SOCs. For instance, the *Angioedema* SMQ contains terms from the *Immune system disorders* SOC (*Systemic allergic reaction, Allergic oedema, Sulfonamide allergy, Type I hypersensitivity*), *Skin and subcutaneous tissue disorders* SOC (*Angioedema, Cholinergic urticaria, Urticaria idiopathic, Acute angio oedema*), *Eye disorders* SOC (*Chemosis, Conjunctival oedema, Edema eyelid*), etc. The size of the SMQs goes from 47 terms (*Scleral disorders SMQ*) up to 8,036 terms (*Malignancies SMQ*). The SMQs can be composed of a flat list of terms or can be hierarchically structured.

### Experimental approach

#### Computing the semantic distance and similarity

Several semantic distance and similarity algorithms can be applied within structured terminological resources [18-21]. In our work, we also rely on this type of approach. In this case, the algorithms count the number of edges (links) between the two terms in order to compute the relatedness of these terms. The simplest algorithm [18] counts the edges between terms and aims to find the shortest path between them. Thus, on the Figure 2, we show an excerpt from a terminological graph. When we compute the shortest path between the nodes *Pharyngeal abscess* and *Abdominal abscess*, we follow the path within the ADR hierarchy and obtain the shortest path equal to four edges. In addition to the path length, other criteria may be taken into account: hierarchical depth of terms [22,23], information content [24], the nearest common parent [25], etc. Besides the computing of the semantic closeness between two terms or words, these algorithms have been used in different contexts such as word-sense disambiguation [22], information retrieval [23,26], gene annotation [27], and terminology enrichment and adaptation [28,29]. A review of the semantic measure and similarity algorithms common within the biomedical area has appeared [30].

In our work, we separately exploit three algorithms to compute the semantic distance and similarity between two terms *t1* and *t2*: (1) the *Rada* semantic distance [18] relies on the computing of the shortest path *sp*; (2) the *LCH* Leacock and Chodorow semantic similarity [20] relies on the shortest path *sp* and on the maximal depth found within the terminology; (3) the *Zhong* semantic distance [23] relies on the absolute depth of terms and on their closest common parent. Semantic distance and similarity are computed between the MedDRA terms but also between the elements of their formal definitions (*D* and *B*) to make the semantic representation of the terms more fine-grained. To illustrate, let’s consider *Abdominal abscess* and *Pharyngeal abscess* terms from Figure 2. The weight of edges is set to 1 because all relations are of the same kind (hierarchical subsumption), and the value of each shortest path corresponds to the sum of weights of all its edges. For this pair of terms we obtain the following shortest paths *sp*: *sp*_*A* *D* *R*_=4, *sp*_*B*_=10 and *sp*_*D*_=0. The unique semantic distance between the MedDRA terms for each semantic distance measure is computed as follows:

$$\forall x\in \{\text{Rada},\text{LCH},\text{Zhong}\},\frac{\underset{i\in \{\text{ADR},D,B\}}{\sum }W_{i}*{\text{sd}}_{x}\left(t1_{i},t2_{i}\right)}{\underset{j\in \{\text{ADR},D,B\}}{\sum }W_{j}},$$

where {*ADR*,*D*,*B*} respectively correspond to the MedDRA ADR terms, and the axes Disorders *D* and Body structure *B*; *t*1 and *t*2 are two MedDRA ADR terms; *W*_*i*_ is the coefficient associated with each of the three axes (the value is set to 1 for *B* and *ADR* and to 2 for *D* to reflect the importance of the latter [31]); and *sd*(*t*1,*t*2) is the semantic distance between *t*1 and *t*2, computed on a given axis with one of the three semantic distance measures *{Rada, LCH, Zhong}*. For the example above, the unique semantic distance is 3.5. According to the tested parameters (three semantic distance measures and MedDRA terms with or without their formal definitions), we build six symmetric matrices with the MedDRA terms from ontoEIM.

#### Term structuring methods

The terminology structuring provides methods for the detection of semantic relations between terms. Two strategies may be distinguished: those which rely on the internal analysis of the terms and those which rely on the contexts within which the terms occur. Because we are working on the terms out of their context, we exploit the terminology structuring methods which rely on the internal analysis of the terms. These methods are applied to a flat list of 18,209 MedDRA PTs. They lead to the detection of hierarchical subsumption and synonymy relations between these terms. The terms are pre-processed: the POS-tagging is done with Genia tagger [32] and the syntactic analysis with the YATE A shallow parser [33]. Three methods are then applied for the acquisition of semantic relations: lexical inclusions, morpho-syntactic variants and compositionality.

##### Lexical inclusion and hierarchy

The basic statement on lexical inclusion hypothesis [34] states that when a given term is lexically included in another term there is a semantic subsumption between them. This hypothesis is well verified in the biomedical area [35,36].

**Figure 3:**
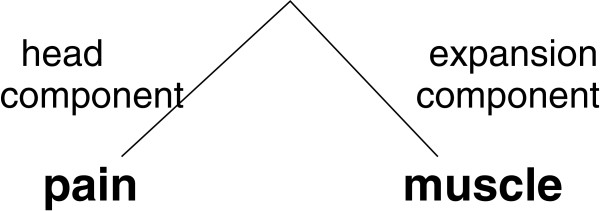
**Syntactic analysis of terms for the induction of hierarchical relations.** Figure 3 presents the syntactic analysis of the term *muscle pain*, its decomposition into head and expansion components, which is then used for the induction of hierarchical relations between this term and its head component.

We distinguish three steps within this approach: 

● the terms are syntactically analyzed into head and expansion components. For instance, on Figure 3, the syntactic analysis of the term *muscle pain* results in two components: head component *pain* and expansion component *muscle*;

● the syntactic and semantic relation is then established between a given term and its head component. For instance, the term on Figure 3 provides the relation between *muscle pain* (the whole term) and *pain* (the head component of the term).

● With these specifications, the identified relations are hierarchical: the long term *muscle pain* is the hierarchical child of the short term *pain*. Indeed, *muscle pain* conveys a more specific information;

● parent and child terms have to be MedDRA terms, otherwise the identified relations are removed.

With the applied specifications of this approach, the identified relations are induced from lexical and syntactic information conveyed by the analyzed terms. Besides, these specifications guarantee that the identified relations correspond to the hierarchical subsumption. In fact, we do not allow the induction of other kinds of relations. For instance, if relations between the whole terms and their expansion components were allowed, the identified relations would be associative, such as localization for example from Figure 3: *muscle pain* is localized in *muscle*.

##### Morpho-syntactic variants

We work with Faster [37] for the identification of morpho-syntactic variants between the *PT* terms. This tool uses several transformation rules, such as insertion (*cardiac disease*/*cardiac valve disease*), morphological derivation (*artery restenosis*/*arterial restenosis*) or permutation (*aorta coarctation*/*coarctation of the aorta*). Each transformation rule is associated with hierarchical or synonymy relations: the insertion introduces a hierarchical relation (*cardiac valve disease* is more specific than *cardiac disease*), while the permutation introduces a synonymy relation. When several transformations are involved, such as in *gland abscess* and *abscess of salivary gland* (combination of permutation (synonymy) and insertion (hierarchy) rules), the hierarchical relation prevails.

##### Compositionality and synonymy

The synonymy relations are acquired in two ways: 

1. The synonymy relation is established between two simple MedDRA terms if this relation is provided by the lexical resources.

2. The identification of synonym relations between complex terms relies on the semantic compositionality [38]. Compositionality appears to be a common characteristics of the biomedical terms [16,39,40].

**Figure 4:**
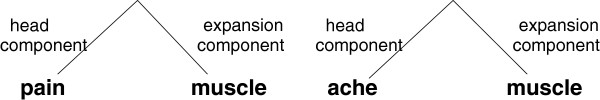
**Syntactic analysis of terms for the induction of synonymy relations.** Figure 4 presents the syntactic analysis of the terms *muscle pain* and *muscle ache*, their decomposition into head and expansion components, which is then used for the induction of synonymy relations between these two terms.

In our work, we consider that two complex terms are synonyms if one of their components at the same syntactic position are synonyms and the other components are identical or also synonyms. For instance, given the synonymy relation between *pain* and *ache* provided by the lexical resources, the terms *muscle pain* and *muscle ache* are also identified as synonyms [41] (Figure 4).

Three transformation rules are applied: on the head component like in the given example, on the expansion component, and on head and expansion components.

We perform several experiments: each medical synonymy resource is used individually and then combined with WordNet.

#### Clustering of terms

During the clustering step, it is important to distinguish between disjoint and non disjoint clusters: with disjoint clusters a given term may belong to at most one cluster, while with non disjoint clusters there is at least one term that belongs to more than one cluster. We give advantage to the non disjoint clusters because they suit better the specificity of our objectives: one MedDRA term may belong to several SMQs.

For clustering the terms on the basis of their semantic distance and similarity, we use two clustering methods: hierarchical ascendant classification *HAC* and Radius *R* method. With *HAC*, the method first chooses the best centers for clusters and then builds the hierarchy of terms by progressively merging the smaller clusters into bigger ones to finally obtain one unique cluster. The obtained dendrogram is then segmented into *k* disjoint clusters. With the *R* radius approach, every MedDRA term is considered as a possible center of a cluster and its closest terms are clustered together with it. This method generates non disjoint clusters.

For clustering of terms with the computed hierarchical and synonymy relations, the relations are considered as directed graphs: the terms are the nodes of the graph while the hierarchical relations are the directed edges. We partition these directed graphs in a way that each directed sub-graph correspond to a set of vertices such as at leat one vertix can reach the others by a directed path. Hence, the generated components are non disjoint clusters. To improve the coverage of these clusters, we add the synonyms: if a term has a synonymy relation with the term from a cluster then this term is also included in this cluster. The initial graph is then augmented with two edges going from and to the synonyms.

Finally, we perform two more steps to deduplicate and merge the clusters: 

● Separately for each method (semantic similarity and terminology structuring), we compute whether smaller clusters are included into larger clusters and we merge those clusters which have at least 80% overlap between them.

● Between the clusters computed by the two methods (semantic similarity and terminology structuring), two clusters provided by these methods and which have at least 80% overlap between them are also merged together.

#### Evaluation of the generated results

We first evaluate the correctness of the generated semantic relations, which is done manually by a computer scientist.

We then perform quantitative and qualitative evaluation of the generated clusters. The quantitative evaluation is performed thanks to their comparison with the SMQs. A cluster is associated to the SMQ with which it has the maximal F-measure. For the setting of the thresholds of the semantic distance and similarity algorithms and their evaluation, we perform a ten-fold cross-validation: the data are partitioned into ten subsets, one subset is used for the setting up the methods while the remaining nine subsets are used for the evaluation. This process is done ten times with a different training subset each time. Three classical measures are then computed: precision *P* (percentage of the relevant terms clustered divided by the total number of the clustered terms), recall *R* (percentage of the relevant terms clustered divided by the number of terms in the corresponding SMQ) and F-measure *F* (the harmonic mean of *P* and *R*). The final evaluation values are computed with the thresholds which provide the best results the most frequently during the cross-validation step. We evaluate the clusters from each method separately and after their merging. A qualitative evaluation is done by a medical expert: we perform a failure analysis of our methods. As for the baseline, we chose the most frequently used approach for the grouping of the MedDRA terms, which relies on the hierarchical structure of MedDRA: the exploitation of the hierarchical subsumption of the PTs through the HLT MedDRA level [7,8,42].

## Results

The 7,629 MedDRA terms from ontoEIM have been processed through the three semantic distance and similarity algorithms. An excerpt from the generated matrices is presented in Table 3: for instance, the distance between *Gastric ulcer* and *Gastrointestinal ulcer* is 1, while the distance between *Gastric ulcer* and *Biopsy tongue* is 10, which reflects the semantics of the terms from these two pairs (the first pair of terms is semantically closer than the second pair). The flat list of 18,209 MedDRA terms has been processed with the terminology structuring methods for the detection of hierarchical and synonymy relations. The results for the terminology structuring methods are presented in Table 4. We can observe that the number of the acquired hierarchical relations reaches up to 4,000. The number of the acquired synonyms is lower (nearly 2,000), while the impact of the WordNet resource is very low (37 and 60 relations). The percentage of the MedDRA PT terms involved in the generated hierarchical relations is 32%. It reaches up to 40% when the synonymy is also considered. With semantic distance, all the terms from ontoEIM, 51% of the MedDRA *PTs*, are used.

**Table 3:** Semantic distance matrix

	**Gastric ulcer**	**Venooclusive liver**	**Reflux gastritis**	**Biopsy tongue**	**Gastrointestinal**
		**disease**			**ulcer**
Gastric ulcer	0	5	3	10	1
Venooclusive liver disease	5	0	7	11	6
Reflux gastritis	3	7	0	12	5
Biopsy tongue	10	11	12	0	11
Gastrointestinal ulcer	1	6	5	11	0

**Table 4:** Acquisition of semantic relations (hierarchical subsumption and synonymy) between the MedDRA terms

***Relationships***	***Methods***	***Number of relations***
Hierarchical relations	Lexical inclusion	3,366
Hierarchical relations	Morpho-syntactic variation with Faster	743
Synonymy relations	Compositionality with 3 biomed terminologies	1,879
Synonymy relations	Compositionality with 3 biomed terminologies and WordNet	1,939
Synonymy relations	Compositionality with the simple UMLS synonyms	190
Synonymy relations	Compositionality with the simple UMLS synonyms and WordNet	227
Synonymy relations	Morpho-syntactic variation with Faster	100

Table 5 indicates the number of clusters and their size according to the strategies and methods (semantic distance, terminology structuring and merging of the results provided by these two methods). This table shows that semantic distance method provides the majority of the clusters, and that number of clusters and their size increase with the merging of the methods (semantic distance and terminology structuring). With the cross-validation, we tested several parameters and determined the best thresholds: with the *Radius* clustering 4 for *Rada*, 4.10 for *LCH* and 0.02 for *Zhong*; with the *HAC* clustering 300 classes. With these thresholds, the number of clusters and their size become larger. We apply these best thresholds to generate the final set of clusters to be evaluated and analyzed by the expert. The impact of the best thresholds on the clusters varies across the SMQs, but the global average results are improved. For the terminology structuring methods the best results are obtained with lexical inclusions, morpho-syntactic variants and synonyms.

**Table 5:** Clustering of terms: number of clusters and their size

***Strategy***	***Clusters of terms***		
	***#clusters***	***Interval***	***Mean***
Semantic distance	7,564	[2; 1,354]	132.67
Terminology structuring	748	[1; 119]	3.82
(hierarchical+synonymy)			
Merging (semantic distance +	7,684	[1; 1,354]	130.75
hierarchical + synonymy)			

The generated semantic relations and clusters have been evaluated via a comparison with the existing SMQs, with the baseline, and through an analysis provided by a medical expert and a computer scientist. The key observations are that the proposed methods outperform the baseline and that the merging of the methods improves the results. We have also observed several limitations of the methods and results. We discuss the performed analysis and evaluations in the following section.

## Discussion

### Limitations of the ontoEIM resource

The ontoEIM resource is unique of its kind, but currently it suffers from incompleteness: only 51% of the MedDRA PTs are aligned with the SNOMED CT terms. The main reason for this is that ontoEIM integrates the alignments between these two terminologies which are already proposed by the UMLS. The integration of additional alignments [43,44] is planned but requires an important expertise of pharmacovigilance experts. Moreover, the recent development of this resource [45] is oriented to the maintenance of the MedDRA hierarchical structure and on some of the existing SMQs. These two points (use of the MedDRA hierarchical structure and description of some existing SMQs) are not suitable for the methods we designed.

### Correctness of the semantic relations

A manual analysis of the generated hierarchical relations, done by a computer scientist, indicates that these relations are usually correct: the syntactic constraints guarantee correct propositions. Nevertheless, we observed a small number of syntactic ambiguities. They appear within 144 pairs (5%) with maximal syntactic heads and correspond to pairs like: {*anticonvulsant drug level, drug level*}, {*blood smear test, smear test*}, {eye movement disorder, movement disorder}. Thus, within the first pair, there is an ambiguity on *drug* as two dependencies seem possible: {*anticonvulsant drug level, drug level*} as proposed with the maximal syntactic head analysis or {*anticonvulsant drug level, level*} (analysis provided with the minimal syntactic head). In our work, we give preference to the syntactic analysis with maximal syntactic heads. But whatever the performed syntactic analysis, the semantic relations remain correct. Nevertheless, we will see that, although the generated semantic relations are deemed correct, the relevance of these relations and of the terms they link is not always perfect to the building of the SMQs. Indeed, some of the terms are seen to be relevant to the SMQs while others do not, which may be due to the difference existing between the linguistically observable semantics of the relations and their domain or medical validity.

### Quantitative evaluation of the generated clusters through their comparison with the SMQs

In Table 6, we indicate the average values of Precision, Recall and F-measure obtained with each method individually (semantic distance and terminology structuring) and when merged. The average precision is usually higher than 45%, although the recall is lower especially with the terminology structuring methods. This is due to the fact that the clusters generated with our methods are smaller than the SMQs and show their different aspects. In this table, we can also see that the merging of the methods allows to improve the average performance of the generated clusters (Recall and F-measure), although we lose one percent in Precision.

**Table 6:** Evaluation results against the gold standard (84 SMQs): average values

***Methods***	***Precision***	***Recall***	***F-measure***
Semantic distance	45.2	32.4	36.9
Terminology structuring	68.4	12.2	18.7
Merging	44.2	36.5	40.0

**Figure 5:**
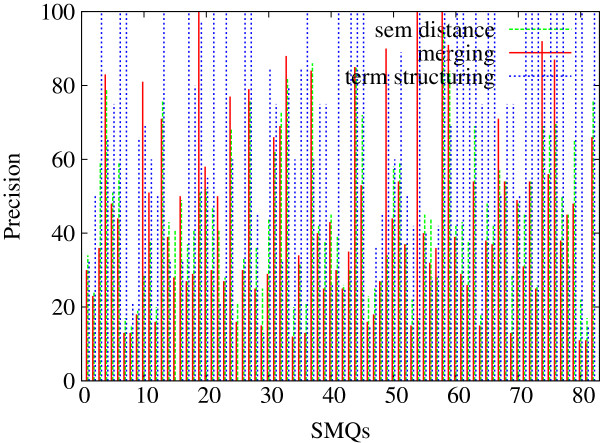
**Evaluation of the clusters generated with the proposed methods (84 SMQs): Precision.** Figure 5 presents the evaluation Precision values obtained further to the evaluation of the clusters generated with the proposed methods (semantic distance, terminology structuring and merging) against the gold standard data (84 SMQs).

**Figure 6:**
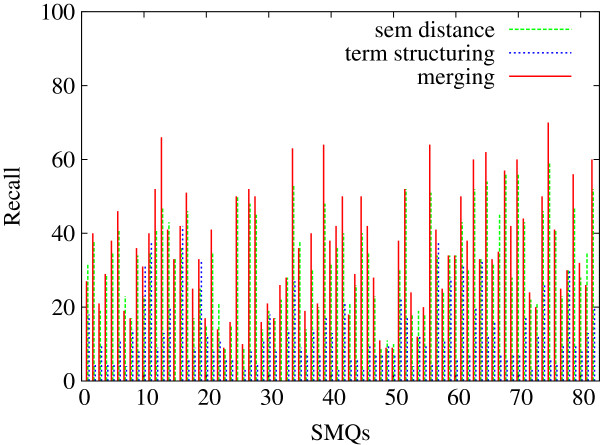
**Evaluation of the clusters generated with the proposed methods (84 SMQs): Recall.** Figure 6 presents the evaluation Recall values obtained further to the evaluation of the clusters generated with the proposed methods (semantic distance, terminology structuring and merging) against the gold standard data (84 SMQs).

**Figure 7:**
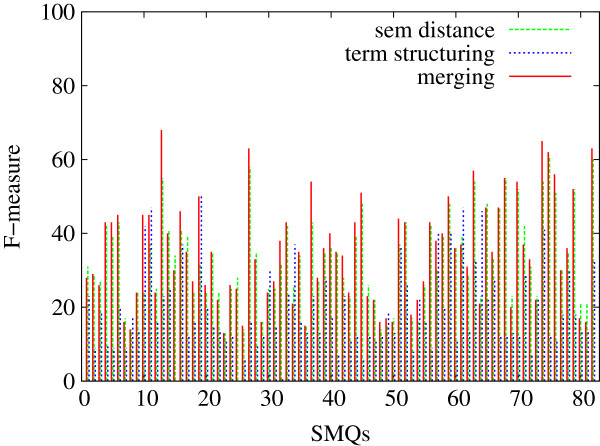
**Evaluation of the clusters generated with the proposed methods (84 SMQs): F-measure.** Figure 7 presents the evaluation F-measure values obtained further to the evaluation of the clusters generated with the proposed methods (semantic distance, terminology structuring and merging) against the gold standard data (84 SMQs).

In Figures 5, 6 and 7, we indicate the evaluation results obtained against all the 84 SMQs for the three evaluation measures: Precision (Figure 5), Recall (Figure 6) and F-measure (Figure 7). Each figure shows the performance of the tested methods (terminology structuring and semantic distance). The *x* axis represents the 84 reference SMQs, the *y* axis the evaluation results. On the whole, we can observe that precision is usually higher than recall, and that there is an important variation across the SMQs. In our previous experiments [46], we gave advantage to precision, while in the current experiment F-measure is advantaged, which improves the global results by eight points. When we look closer at these Figures, we can observe for example that, in Figure 5, the terminology structuring method has the highest precision (with over 30% of the clusters showing 100% precision), while the semantic distance method shows the lowest precision. The situation is different with recall in Figure 6: the terminology structuring method has the lowest values, while the semantic distance method has the highest values. In Figure 7, we can see that the merging of the methods very often outperforms the semantic distance methods. These figures also point out that there is a great variability across the SMQs, while currently we use the same setting of the methods independently from the SMQs.

We also performed an additional analysis of the clusters generated with the terminology structuring which shows the following contribution of the generated semantic relations: 

1. the hierarchical relations form the basis of the clusters: they correspond to 96% of the involved terms and show 69% precision. Only three clusters do not contain hierarchical relations;

2. the Faster relations are involved in 50% of clusters and show a precision between 75 and 85%;

3. one third of the clusters contains synonymy relations, and their precision varies between 55 and 69%;

4. the relations acquired with the UMLS resources are involved in 14% of clusters while their precision is only 38%;

5. the WordNet-based relations involve only six terms (such as those involved in the relations {*heart syndrome, nerve degeneration*} and {*heart injury, nerve damage*}). The whole impact of the WordNet synonyms is almost null. Moreover, the involved terms are either proposed by other more contributory methods or do not correspond to correct propositions. The most interesting (and correct) relation is {*intestinal gangrene, gastrointestinal necrosis*}. It is unique to the WordNet resource output.

On the whole, observations of the impact of the methods and resources on their contribution correspond to the expected results but provide also with surprises. Thus, the highest precision is observed with the morpho-syntactic Faster relations: these are based upon the morphological variations of the terms and usually convey minor semantic modifications ({*abdomen, abdominal*}, {*infect, infection*}). The synonymy relations may involve greater semantic variations (such as in {*sepsis, infection*} or {*abdominal, intestinal*}) and this explains their less impressive but still acceptable precision. Moreover, the synonym terms may have a contextual semantic value [47], *i.e.* be valid in some but not in all the contexts. As a matter of fact, the pairs {*sepsis, infection*} and {*abdominal, intestinal*} have been acquired from terms considered as synonyms in the existing terminologies. However, these synonyms are not deemed to be correct for the creation of the SMQs. Finally, the hierarchical relations convey yet greater semantic variation (the hierarchical child terms are semantically more specific than the parent terms), although their precision is higher than the precision of the synonym relations. Moreover, the generated hierarchical relations participate very actively in the creation of the clusters of terms. As we previously observed, the generated hierarchical relations bring the majority of terms in the clusters. This is a surprising observation: we did not expect to receive such a great contribution from the hierarchical relations. Another surprising observation is related to the poor contribution of the synonymy relations from WordNet and those extracted directly from the UMLS: both their coverage and precision are weak and they are weakly involved in the creation of the clusters.

### Comparison with the baseline

**Figure 8:**
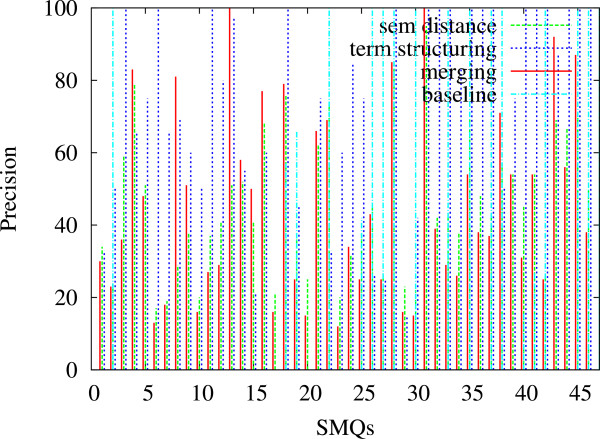
**Comparison of the generated clusters with the baseline (46 SMQs): Precision.** Figure 8 presents the precision values obtained further to the comparison of the baseline with the proposed methods: exploitation of the MedDRA hierarchical structure and of the hierarchical subsumption of the PT terms through their HLT terms. We consider the 46 SMQs which have equivalent HLT terms.

Our baseline is the most common method utilized for the grouping of the PT terms within MedDRA: their hierarchical subsumption through the HLT terms. Among the 1,688 HLTs and 84 SMQs, 46 of them have either direct (*Thrombocytopenias (SMQ)* and *Thrombocytopenia (HLT)*) or non ambiguous correspondences (*Renal failure and impairment (SMQ)* and *Acute renal failure (HLT)*). We use these 46 SMQs as gold standard with the baseline hierarchical subsumption and with our methods. These 46 SMQs are a subset of the whole set of the 84 SMQs. Similarly to the results presented in the previous paragraph, the average performance on the baseline set is indicated in Table 7, while the detailed performance per evaluation measure is indicated on Figures 8 (Precision), 9 (Recall) and 10 (F-measure). We can observe that on average, the baseline approach can be compared with the terminology structuring method, although the baseline performance is lesser. The comparison with other experiments points out that precision is higher with the baseline (although very close with the one provided by the semantic distance), while recall and F-measure are notably improved with other methods. The figures also show that the proposed methods outperform the baseline. These are positive observations which clearly indicate that the proposed methods contribute to the state of the art.

**Table 7:** Evaluation results for the baseline and the proposed methods (46 SMQs): average values

***Methods***	***Precision***	***Recall***	***F-measure***
Baseline	60.3	9.2	14.9
Semantic distance	46.0	33.9	34.1
Terminology structuring	71.1	11.8	18.9
Merging	41.0	45.6	37.3

**Figure 9:**
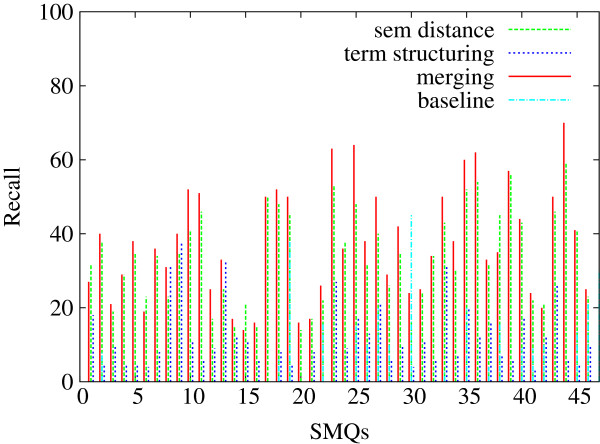
**Comparison of the generated clusters with the baseline (46 SMQs): Recall.** Figure 9 presents the recall values obtained further to the comparison of the baseline with the proposed methods: exploitation of the MedDRA hierarchical structure and of the hierarchical subsumption of the PT terms through their HLT terms. We consider the 46 SMQs which have equivalent HLT terms.

**Figure 10:**
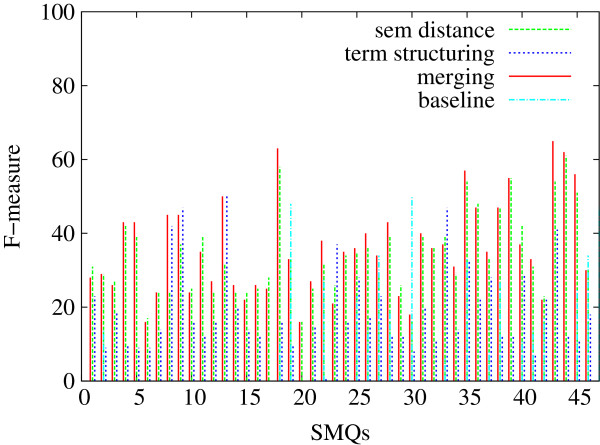
**Comparison of the generated clusters with the baseline (46 SMQs): F-measure.** Figure 10 presents the F-measure values obtained further to the comparison of the baseline with the proposed methods: exploitation of the MedDRA hierarchical structure and of the hierarchical subsumption of the PT terms through their HLT terms. We consider the 46 SMQs which have equivalent HLT terms.

### Qualitative evaluation with an expert

In Table 8, we indicate examples of seven clusters: *Angioedema*, *Embolic and thrombotic events, arterial*, *Haemodynamic oedema, effusions and fluid overload*, *Periorbital and eyelid disorders*, *Peripheral neuropathy*, *Haemolytic disorders* and *Agranulocytosis*. This table indicates the number of terms in the SMQs and in the corresponding clusters (*clu*), as well as the number of common terms between them (*com*) and the performance (Precision *P*, Recall *R* and F-measure *F*) when computed against the reference data *Reference* and also after the manual analysis performed by the expert (*Manual*). The results obtained with different strategies are indicated: the semantic distance *sd*, the terminology structuring *struc*, as well as the merging *merg* of semantic distance and terminology structuring. We can observe that the performance of the methods varies a lot across the presented SMQs and clusters. Usually, the terminology structuring provides a higher precision and lower recall than the semantic distance measures. The semantic distance and merging approaches generate bigger clusters: they lead to increased recall but they decrease the precision. Usually, the F-measure takes advantage and is improved. The manual evaluation by the expert accepts additional terms, which allows to have a more complete picture of the performance of the proposed methods. This expert evaluation leads also to increased precision, recall and F-measure.

**Table 8:** Evaluation results against the gold standard and further to the manual analysis of the expert

***SMQs***	***Number of terms***			***Reference***			***Manual***		
	***SMQ***	***clu***	***com***	***P***	***R***	***F***	***P***	***R***	***F***
*Angioedema*_*s* *d*_	52	56	20	36	38	37	41	44	42
*Angioedema*_*s* *t* *r* *u* *c*_	52	31	19	61	36	45	61	36	45
*Angioedema*_*m* *e* *r* *g*_	52	41	21	51	40	45	71	48	57
*Embolic and thrombotic events...*_*s* *d*_	132	140	48	34	36	35	39	41	40
*Embolic and thrombotic events...*_*s* *t* *r* *u* *c*_	132	13	12	92	9	16	92	9	16
*Embolic and thrombotic events...*_*m* *e* *r* *g*_	132	140	48	34	36	35	47	46	46
*Haemodynamic oedema, effusions...*_*s* *d*_	36	56	13	23	36	28	38	58	50
*Haemodynamic oedema, effusions...*_*s* *t* *r* *u* *c*_	36	31	13	42	36	39	84	72	78
*Haemodynamic oedema, effusions...*_*m* *e* *r* *g*_	36	41	15	37	42	39	81	92	86
*Periorbital and eyelid disorders*_*s* *d*_	39	44	22	50	56	53	52	59	55
*Periorbital and eyelid disorders*_*s* *t* *r* *u* *c* *t*_	39	4	4	100	10	18	100	10	18
*Periorbital and eyelid disorders*_*m* *e* *r* *g*_	39	45	22	48	56	52	78	46	58
*Peripheral neuropathy*_*s* *d*_	31	58	16	27	51	36	45	84	59
*Peripheral neuropathy*_*s* *t* *r* *u* *c* *t*_	31	2	2	100	6	12	100	6	12
*Peripheral neuropathy*_*m* *e* *r* *g*_	31	58	16	28	52	36	60	80	69
*Haemolytic disorders*_*s* *d*_	26	27	12	44	46	45	66	69	67
*Haemolytic disorders*_*s* *t* *r* *u* *c* *t*_	26	3	3	100	11	20	100	11	20
*Haemolytic disorders*_*m* *e* *r* *g*_	26	27	12	44	46	45	78	81	79
*Agranulocytosis*_*s* *d*_	29	25	7	28	24	26	32	27	29
*Agranulocytosis*_*s* *t* *r* *u* *c*_	29	13	9	69	31	42	77	34	47
*Agranulocytosis*_*m* *e* *r* *g*_	29	11	9	81	31	45	77	34	47

We performed a detailed qualitative analysis of seven SMQs and clusters with the medical expert.

For instance, the SMQ *Angioedema* contains 52 terms which mean signs and symptoms of angioedema. The semantic distance algorithm provides a cluster with 56 terms, among which 36 do not belong to this SMQ. Three of them (*Injection site urticaria*, *Cervix oedema* and *Injection site swelling*) could be included in the SMQ because they are caused by drugs and are indeed the symptoms of angioedema. Eight more terms (*i.e.*, *Solar urticaria*, *Urticaria thermal*, *Urticaria contact*) are true false positives because they are not related to angioedema. Finally, other terms, although they mean oedemas, are not caused by drugs. Thus, according to the expert, three of the 36 false positives should be considered for the inclusion in the SMQ. As for the terminology structuring method, it provides a cluster with 31 terms, among which 12 do not belong to the SMQ. According to the expert, this evaluation is correct: the term *Injection site oedema* has a too broad meaning (although this SMQ seems to contain other broad terms, such as *Gingival injury* and *Skin lesion*), while 11 other terms mean oedemas not caused by drugs. With the merging we improve the performance: we obtain two more true positives (*Oedema peripheral*, *Generalized oedema*), while the false positives remain the same. The results are different because the merging is not supervised (it is based upon the intersection between the clusters): the clusters may be different when considered separately for each method and when considered through their merging. As a matter of fact, this is precisely what happens with the *Angioedema* SMQ: during the merging step, the clusters selected are different from those selected during the evaluation of the individual methods, and we gain one new true positive term.

For the SMQ *Embolic and thrombotic events*, our methods provide 92 false positives with the semantic distance and one with terminology structuring. The analysis of these terms is very similar to what we observe for other SMQs: some of the proposed terms should be considered for inclusion in the SMQ (such as *Iliac artery stenosis, Hepatic artery stenosis, Vertebral artery stenosis, Cerebellar ataxia, Penile vascular disorder*) because they are very close to the already included terms, other terms have a too broad meaning to efficiently contribute to the SMQ (*Peripheral ischaemia*, *Chest injury*, *Ischaemia* or *Infarction*). Finally, some other terms (*Mesenteric artery stenosis*...) are true false positives. Among the false negatives of the *Angioedema* SMQ, we find terms such as *Wheezing, Drug hypersensitivity, Swollen tongue, Penile oedema*, and among the false negatives of the *Embolic and thrombotic events* SMQ, we find terms such as *Venous occlusion, Splenic infarction, Subclavian artery thrombosis*. The main reasons of the false negatives are: (1) with the semantic distance and similarity algorithms, in addition to the fact that only 51% of the MedDRA terms are included in the ontoEIM resource, when the terms are included they may be too distant in this resource, (2) with the terminology structuring, the methods may be not exhaustive enough to detect all the lexical and syntactic regularities within the terms.

## Conclusions and perspectives

We combined several strategies and methods for the clustering of the MedDRA terms with similar or close meaning. We performed a comparison of the results obtained and analyzed their complementarity. A ten-fold cross-validation was carried out in order to test different parameters and select those which positively influence the results. Although the automatic creation of the SMQs is a difficult task, our results indicate that the automatic methods may provide a basis for the creation of the SMQs. The current evaluation has been done against the existing SMQs, but we expect we can apply the same method for the creation of new SMQs with similar performance. Our methods generate clusters which are smaller than the corresponding SMQs and which show their different aspects. For this reason, the precision of the clusters is often high, while their merging leads to the improvement of their completeness.

Our future work will address the current limitations of our methods and results. The material, and more particularly the ontoEIM resource, is being improved thanks to a better alignment with the SNOMED CT [43,44]. Moreover, the future studies will lead to the identification of other parameters which influence the quality of clusters and also of other factors and more robust methods for the merging of clusters [48-50]. Also, we would like to address the points related to the complementarity of the clusters and their potential hierarchical dependencies. As we observed, the performance varies according to the SMQs and it appears that different strategies should be used for different SMQs, while currently we apply the same settings of the methods to all the SMQs. We plan to perform an exhaustive analysis of the nature of semantic relations which can be observed within the SMQs, which will allow to propose other methods and to reduce the current false negatives within the clusters. An alternative method will consist into the exploitation of corpora for the detection of other semantic relations among the terms. In addition, we intend to carry out a more detailed evaluation of the generated clusters. This addresses particularly the impact of the generated clusters on the exploring of the pharmacovigilance databases (such as the FDA database) and on the signal detection tasks. The very first results of this type of evaluation (not presented in the article) are very promising because they lead to an improvement of the signal detection by comparison with the results obtained with the SMQs.

## Competing interests

The authors declare that they have no competing interests.

## Authors’ contributions

MD implemented and performed the semantic distance and similarity computing, the clustering experiences and the quantitative evaluation; LD had the responsibility to evaluate the obtained clusters; TH adapted the terminology structuring methods, generated the results with these methods and evaluated their correctness; NG led the work, proposed the methodology, participated in the evaluation and designed the article. All the authors participated in the writing of the article. All authors read and approved the final manuscript.
